# Diagnosing hospital bacteraemia in the framework of predictive, preventive and personalised medicine using electronic health records and machine learning classifiers

**DOI:** 10.1007/s13167-021-00252-3

**Published:** 2021-08-31

**Authors:** Oscar Garnica, Diego Gómez, Víctor Ramos, J. Ignacio Hidalgo, José M. Ruiz-Giardín

**Affiliations:** 1 Departamento de Arquitectura de Computadores, Universidad Complutense de Madrid, Madrid, Spain; 2 Universidad Complutense de Madrid, Madrid, Spain; 3 Departamento de Medicina Interna, Hospital Universitario de Fuenlabrada, Madrid, Spain

**Keywords:** Predictive, Preventive and personalised medicine (PPPM/3PM), Machine learning, Modelling, Bacteraemia diagnosis, Bacteraemia prediction, Blood culture’s outcome prediction, Individualised electronic patient record analysis, Personalised antibiotic treatment, Support vector machine, Random forest, K-Nearest neighbours, Healthcare economy, Health policy, COVID-19

## Abstract

**Background:**

The bacteraemia prediction is relevant because sepsis is one of the most important causes of morbidity and mortality. Bacteraemia prognosis primarily depends on a rapid diagnosis. The bacteraemia prediction would shorten up to 6 days the diagnosis, and, in conjunction with individual patient variables, should be considered to start the early administration of personalised antibiotic treatment and medical services, the election of specific diagnostic techniques and the determination of additional treatments, such as surgery, that would prevent subsequent complications. Machine learning techniques could help physicians make these informed decisions by predicting bacteraemia using the data already available in electronic hospital records.

**Objective:**

This study presents the application of machine learning techniques to these records to predict the blood culture’s outcome, which would reduce the lag in starting a personalised antibiotic treatment and the medical costs associated with erroneous treatments due to conservative assumptions about blood culture outcomes.

**Methods:**

Six supervised classifiers were created using three machine learning techniques, Support Vector Machine, Random Forest and K-Nearest Neighbours, on the electronic health records of hospital patients. The best approach to handle missing data was chosen and, for each machine learning technique, two classification models were created: the first uses the features known at the time of blood extraction, whereas the second uses four extra features revealed during the blood culture.

**Results:**

The six classifiers were trained and tested using a dataset of 4357 patients with 117 features per patient. The models obtain predictions that, for the best case, are up to a state-of-the-art accuracy of 85.9%, a sensitivity of 87.4% and an AUC of 0.93.

**Conclusions:**

Our results provide cutting-edge metrics of interest in predictive medical models with values that exceed the medical practice threshold and previous results in the literature using classical modelling techniques in specific types of bacteraemia. Additionally, the consistency of results is reasserted because the three classifiers’ importance ranking shows similar features that coincide with those that physicians use in their manual heuristics. Therefore, the efficacy of these machine learning techniques confirms their viability to assist in the aims of predictive and personalised medicine once the disease presents bacteraemia-compatible symptoms and to assist in improving the healthcare economy.

## Introduction

### The paradigm shift from reactive to predictive, preventive and personalised medicine

Current best healthcare practices promote the assumption of a predictive medicine tailored to the patient under the Predictive, Preventive and Personalised Medicine (PPPM/3PM) paradigm that is based on, among others, the capacity to predict disease development and influence decisions about lifestyle choices or to customise the medical practice to the patient [1]. Many of these diseases can be accompanied by severe complications. Hence, applying machine learning techniques on the available patient’s data in the electronic hospital records to predict the presence of complications is an example of practical multidisciplinary implementation of PPPM/3PM strategies to improve healthcare.

One of these complications that result in increased morbidity and mortality [2] is bacteraemia. The related in-hospital case-fatality rate in bacteraemia is 12% in some reports [3]. Sepsis is one of the most important causes of morbidity and mortality. It is estimated at 19 million cases, and up to 5 million sepsis-related deaths annually [4].

Machine learning (ML) techniques will contribute an important added value to the three pillars of 3P medicine. Thus, the prediction of this kind of infection is useful either (i) to prevent it or (ii) to decrease its morbidity and mortality by starting an early, appropriate and specific antibiotic treatment. It is recommended that antibiotic treatment be promptly administered whenever there is a suspected serious bacterial infection [5, 6] and, if possible, after blood cultures have been taken. The diagnosis can take up to 6 days using blood cultures which introduces a significant lag in the antibiotic treatment. The individual prediction of bacteraemia would reduce this diagnosis lag enabling the early administration, up to 6 days earlier, of a personalised antibiotic treatment that would significantly reduce the bacteraemia complications.

Additionally, ML techniques can also provide an important added value to the targeted prevention of bacteraemia by identifying patients with bacteraemia and their specific bacteraemia’s source earlier. The bacteraemia’s source determines (i) the specific and most appropriate antibiotic treatment, (ii) the specific diagnostic techniques to search the reasons for the bacteraemia source, and (iii) it helps determine additional treatments that sometimes must be combined with the antibiotic treatment, for example, surgery [7]. In this sense, preventative methods have been shown to be successful, for example, methods such as vaccination or the Michigan-keystone project to reduce central-line related bloodstream infections in children [8].

A personalised and specific antibiotic treatment follows the prediction of bacteraemia and its source. Personalised treatment means that each patient, with its own bacteraemia’s focus and clinical situation (i.e. type of bacterial infection, source of infection, hemodynamic situation, temperature, laboratory markers, age, vaccination coverage, exposure to invasive procedures, if the patient has received antibiotics before, if he has suffered previous hospital incomes, or if a multiresistant microorganism has colonised him), needs a specific antibiotic treatment. All these factors determine the kind of antibiotic that the patient should receive [9, 10] which is intimately related to the morbidity and mortality of the patient.

ML techniques can consider all the previous variables to predict bacteraemia, prevent its complications and help personalise the treatments.

### Bacteraemia

Bacteraemia is the presence of bacteria in the bloodstream [11]. In healthy patients, the blood does not contain bacteria, so its presence is associated with infections that can impact the patient’s life.

The most typical origin for bacteraemia is an infection, restricted to a specific location in the body, that favours the bacteria’s movement into the blood. The most frequent bacteraemia-producing infections are urinary (*prostatitis* or *pyelonephritis*), respiratory (*pneumonia*), vascular (infected catheters), digestive (*cholecystitis* or *cholangitis*), skin and soft tissues (*cellulitis* or *myositis*), or bones (*osteomyelitis*). When the origin is unknown, it is referred to as primary or idiopathic bacteraemia. Some medical procedures can also favour bacteria’s passage into the blood in previously healthy patients, from sites usually colonised by bacteria, such as urinary catheters in the bladder or endoscopies of the digestive tract (colonoscopies). Likewise, certain habits such as intravenous drug use can favour the passage of bacteria from the skin to the blood [12].

The bacteria in the blood can spread the infection to other places in the body, producing *endocarditis*, *arthritis*, *osteomyelitis*, *meningitis*, or brain abscesses, among others. In [13], the authors describe the connection between the type of bacteraemia microorganism and the site of acquisition with associated mortality. They show that the mortality associated with bacteraemia ranges from 11 to 37% depending on the place and type of microorganism. There is a high mortality rate associated with bacteraemias [14], and blood cultures are the gold standard for testing for the diagnosis of bloodstream infections. Due to the high morbidity and mortality associated with bacteraemia, it is mandatory to initiate effective antibiotic treatment as soon as possible to reduce the death rate [15].

Therefore, as presented above, bacteraemia can be either the origin or the complication of diseases on which the PPPM/3PM [16] and personalised medicine [17] are focused on, and the very same principles that guide PPPM can be used to predict the complications’ development and to customise their medical practice.

### Deficits in the current treatment of bacteraemia

The means of detecting bacteraemia is via blood cultures [18, 19] in vials that contain growth media of two types: aerobic and anaerobic. To this aim, an amount of the patient’s blood—from 20 to 40ml—is drawn and introduced into the vials. Then the vials are placed within a system that maintains the optimal environmental conditions (temperature, humidity, light) for the microorganism’s growth. The microorganism’s growth produces CO_2_, and the system detects its production. This process can take between hours and 5 days. If the system does not detect CO_2_ production during this time frame, it reports a negative culture (no bacteraemia), whereas if it does detect CO_2_ production, then it reports a positive culture. Nevertheless, a positive culture does not always imply bacteraemia. Therefore, it is also important to determine if this growth is a true bacteraemia or a contaminant (negative bacteraemia). If a positive culture appears, then the identification of the microorganism, the bacteria species that have grown in the vials, begins. The complete process of identifying the microorganism can take up to another 2 to 3 days. In many cases, the species identified came from the skin or was introduced in the blood sample either during blood extraction or during the culture. In such a case, the culture is contaminated and considered to have no bacteraemia. Finally, only those analyses in which the bacteria species comes from an infection are declared to be bacteraemia.

The prediction of true bacteraemia has two important moments. The first one is when the physician decides to extract blood from the patient for the blood culture. The second one is the moment (hours or days after the blood extraction) when some blood cultures are positive. From this second moment to the definitive identification of the microorganism can take 2 or 3 days. Among these positive blood cultures (i.e. the system detects CO_2_), some will be contaminants (considered to be negative bacteraemia), and others will be true cultures (considered to be true bacteraemia). The type of blood culture (aerobic or anaerobic blood cultures) and the time lapse to detect growth could be important to predict if the growth is true or not in this second period, before the definitive identification of the microorganism.

The deficits in the current treatment of bacteraemia begin at the moment that it is decided to obtain blood cultures. Blood cultures should not be obtained indiscriminately because this increases the number of contaminated blood cultures, leading to unnecessary antibiotic therapy and increasing economic costs. There are different situations in which blood cultures should be obtained, such as severe sepsis, suspected infection with organ dysfunction, high blood lactate levels, or infectious processes associated with bacteraemia (for example, *pyelonephritis*, *cholangitis*, severe pneumonia, *meningitis*, suspected *endocarditis*, or endovascular infections). Also, bacteraemia should be suspected in patients with fever and at least one other sign or symptom of infection in the absence of a known alternative diagnosis.

For the physician, it is important to predict bacteraemia before deciding to obtain blood cultures. Unfortunately, physicians are not good at predicting which patients have bacteraemia [20]. The result of this poor prediction of bacteraemia is a low rate of true positive blood cultures; [21] reports rates between 5 and 10% and [22] reports values as low as 3.6% per analysis.

The second point regarding deficits in the current treatment of bacteraemia is the interpretation of positive blood cultures. There are organisms that should never be considered contaminants when identified in blood cultures, such as gram-negative roads, *Staphylococcus aureus*, or *Candida* spp. On the other hand, organisms such as coagulase-negative *Staphylococcus* spp. and *Corynebacterium* sp. are usually common skin contaminants, and if they are obtained in blood cultures, they usually do not need antibiotic treatment. However, sometimes this last group, usually contaminants, could produce bacteraemia mostly related to catheters or prosthetic valves.

The items explained above are related to the decision regarding antibiotic treatment and how long a patient should be treated. Therefore, predictive models of bacteraemia could help the physician make the appropriate decision regarding these points. Thus, in this sense, PPPM/3PM has a very important point of intervention in suspected bacteraemia and its treatment.

### Clinical, economic and structural consequences

The usefulness of blood cultures in predicting bacteraemia is low, with a range between 4.1 and 7% [21, 23]. Compared to the true positive rate, false positive results due to contamination are in a similar or a higher range, varying between 0.6 and over 8% [24]. These problems of blood culture analysis also have an important economic impact, with a 20% increase of total hospital costs for patients with false positive blood cultures [25, 26]. Economic analyses estimate the costs related to a single false positive blood culture can be between $6878 and $7502 per case [24, 27]. In 2012, the American Board of Internal Medicine introduced the Choosing Wisely campaign, which aimed to reduce medical waste and the overuse of blood cultures by setting clear guidelines for the use of blood cultures. Studies assessing risk factors for bacteraemia have led to the development of multiple stratification systems without consensus [28].

### State of the art

Specialised prediction models can help make clinical decisions. The goal is to provide patient risk stratification to support tailored clinical decision-making. Clinical prediction models use variables selected because they are thought to be associated (either negatively or positively) with the outcome of interest [29]. On the other hand, risk prediction models can be used to estimate the probability of either having (diagnostic model) or developing a particular disease or outcome (prognostic model) [30].

Regarding prediction models for bacteraemia, a physician’s suspicion of bacteraemia lacks sensitivity, specificity, or predictive values to be clinically useful. Some examples of clinical prediction models have been developed with bacteraemia related to pneumonia [31, 32], skin infections [33], and community-acquired bacteraemias [34]. Unlike ours, they all are focused on specific infections, which applies to any source of intra- or extra-hospital bacteraemia. In addition, none of them uses ML techniques, but rather methodologies ranging from multivariable analysis to identify significant predictors for bacteraemia [31], stepwise logistic regression, or multiple mutually exclusive stepwise logistic regression.

To the best of our knowledge, there is no application of ML techniques to create diagnostic bacteraemia models. Nevertheless, ML has had a successful history in biomedicine with applications in almost all the facets of medicine [35]: neural networks for breast cancer diagnosis [36], bladder cancer [37] or colorectal cancer [38], ensemble classifiers in bioinformatics [39], deep residual networks for carcinoma subtype identification [40], Tree-Lasso logistic regression [41], Bayesian Networks [42] for the prediction of the causative pathogen in children with osteomyelitis or decision trees [43] to cite just a few recent examples. Regarding classifiers, recently they have been used for cancer diagnosis using K-Nearest Neighbours (KNN) [44], drug identification using Support Vector Machine (SVM) [45] or predicting risk of disease using Random Forest (RF) [46], again to cite some illustrative examples in a myriad of papers.

### Working hypothesis

For the aforementioned reasons, it would be interesting to predict which patients suffer from this pathology before deciding on blood sample extraction, and if the physician has decided to obtain blood cultures, it would be of interest to predict which patients will suffer true bacteraemia without waiting for up to 6 days for the definitive results. There are no useful clinical, analytical or epidemiological studies that allow physicians to predict bacteraemia at the patient’s initial assessment.

Hence, our work’s main objective is to implement ML techniques on a set of patient data from electronic hospital records to predict the appearance of bacteraemia, thus eliminating the wait for the results of blood cultures and anticipating the application of therapeutic treatments. Three ML techniques have been used: SVM, RF and KNN. The potential of these models in terms of PPPM/3PM is that used in conjunction with clinical judgement, they can be useful in the decision-making process regarding blood culture collection, clinical monitoring and empirical antimicrobial therapy. This work could provide two benefits: first, the possibility of starting the personalised patient’s treatment earlier; second, the number of blood cultures would be reduced since they would only be prescribed in cases where the techniques’ predictions did not have high reliability.

The rest of the paper is structured as follows. Section “Materials and methods” is devoted to introducing the material and methods of this study. Next, Section “Data analysis” presents the data analysis, Section “Discussion of the results” discusses the findings, and, finally, Section “Conclusions and recommendations in the framework of 3P medicine” summarises the conclusions and presents the recommendations in the framework of 3P medicine.

## Materials and methods

### Subject database

The database is provided by the Hospital Universitario de Fuenlabrada, Madrid, Spain, a 350-bed hospital with the following services: general surgery, urology, orthopaedic surgery, gynaecology and obstetrics, paediatrics, intensive care units (ICUs), haematology-oncology, internal medicine and cardiology. The database was gathered from 2005 to 2015, and it consists of 4357 anonymous patient records, a.k.a. instances, containing 117 features per patient, 49.3% female with age 65.1 ± 19.7, and 56.1% male with age 62.7 ± 20.2. Each instance contains demographic and medical data (medical history, clinical analysis, comorbidities, etc.) and the result of the blood culture, the feature to be predicted, which can take one of two values: bacteraemia and no bacteraemia. The database contains 2123 bacteraemia (51.3%), which includes aerobic, strict anaerobic and facultative anaerobic bacteria, and 2234 no bacteraemia (48.7%), including 1844 contaminations.The final classification of true bacteraemia was done in prospective time by an infectious disease physician, using all the previous data, including microbiological, clinical and analytical data.

Forty-seven out of the 117 features were discarded from the database because they are derived from other features, irrelevant to the study, or useful after the blood culture was identified.

Two datasets were created from the database. The first dataset, called pre_culture, only uses the features known previously to the blood culture, i.e. the ML techniques only use the 65 features available previous to the culture to predict the bacteraemia, having discarded the features that hold the suspected source of infection. The second dataset, called mid-culture, uses the data available when the concentration of CO_2_ starts rising. Note that, as stated in “Introduction”, an increase of CO_2_ could be either due to a true bacteraemia or a contamination of the blood sample during extraction, so the increase of CO_2_ does not necessarily mean bacteraemia. In this sense, contamination has the same value as no bacteraemia. The number of features in this dataset is 69: the 65 features in pre-culture plus four new ones: the time to CO_2_ detection, the type of media with bacterial growth, either aerobic or anaerobic and the first vial where the growth is detected (see “Appendix A: Features in the study” for an enumeration of the features under study).

### Data preprocessing

#### Categorical features

Both datasets contain a set of patient instances, $\mathcal {P}_{i}$, so that every instance comprises the medical (microbiological, clinical and analytical) and demographic data of one patient. $\mathcal {P}_{i}$ is the concatenation of a feature vector, **f**_*i*_, and the classification—predicted—variable, *y*_*i*_, that is $\mathcal {P}_{i} = (\mathbf {f}_{i},y_{i})$. **f**_*i*_ defined on a feature space, $\mathbb {F}$, of dimension *L*, $\mathbb {F} = F^{1} \times F^{2} \times {\ldots } \times F^{L}$ so that each *F*^*i*^ is the set of values of a medical or demographic feature of the patient, i.e. age, fever, comorbidities, etc., and *y*_*i*_ ∈{− 1,1} is the result of the blood culture, either ‘1’ when the patient has bacteraemia or ‘− 1’ when he or she does not. Therefore, $\mathbf {f}_{i} =\left ({f_{i}^{1}} \in F^{1}, {f_{i}^{2}} \in F^{2}, \ldots , {f_{i}^{L}} \in F^{L} \right )$ and the datasets are $ \left \{ \mathcal {P}_{i} = (\mathbf {f}_{i},y_{i}) \mid \mathbf {f}_{i} \in \mathbb {F}, y_{i} \in \left \{ -1,1 \right \} \right \}$.

SVM and KNN require a definition of distance on $\mathbb {F}$. This requirement imposes the categorical features to be translated into numerical values. However, the mapping of categorical values onto numerical ones without detailed supervision will bias the ML algorithm because the numerical translation will define proximity relationships that are not present in the categorical feature. The most used codification to avoid these problems is the one-hot encoder. It loops through the dataset and separates each feature of a given categorical type into subcategories; that is, for each category in a feature, the technique generates a new feature with only two values: true or false. Consequently, this technique defines a new feature space, $\mathbb {F}'$ with a number of features $L^{\prime }$. On $\mathbb {F}'$, the distance metric, $d: \mathbb {F}' \times \mathbb {F}' \xrightarrow {} \mathbb {R}$, can be defined now. The Euclidean distance, given by Eq. (1), was chosen.

1 $$  d(\mathcal{P}_{i}, \mathcal{P}_{j}) = \sqrt{{\sum}_{d=1}^{L^{\prime}}{({f_{i}^{d}} - {f_{j}^{d}})^{2}}}   $$

#### Missing data

The method to handle missing data depends on the nature of the data missingness. Three categories have been defined to classify missingness [47]: (i) missing completely at random (MCAR) in which the missingness is random, unrelated to the outcomes and does not contain valid information for analysis; (ii) missing at random (MAR) when the missingness depends on the outcomes observed; and (iii) missing not at random (MNAR) when missingness depends on unobserved measurements.

To check the missingness of the data, we define, one feature at a time, two classes, missing and non-missing data, a RF classifier is built upon this feature, and we evaluate if the missing data provides a good classification using the RF classifier [48]. If RF accuracy is high for this feature, a MAR behaviour is concluded for the feature and discard it from the dataset.

Three different approaches are evaluated to handle the high number of missing data [49]. The complete case data approach removes the instances with missing data to obtain a new dataset without misses; that is, all instances have valid data in all features. This approach presents two handicaps: (i) its usage would not allow a new instance with missing data to be evaluated once the ML model is trained and tested, and (ii) it significantly reduces the dataset.

An alternative approach that attempts to keep a large ratio of complete instances in the dataset is also evaluated [50]. This method ranks the features in decreasing order in the percentage of missing data and then iteratively removes the features following the ranking order. In each iteration, the number of complete instances is calculated and the total quantity of data in the complete instances, i.e. the number of complete instances times the number of instances. As the number of features decreases, the total amount of non-missing data in the complete instances increases to a maximum, beyond which the quantity of non-missing data in complete instances decreases. This maximum determines the number of features that most contribute to complete case instances, and it is the best option.

Both previously mentioned methods operate under the MCAR supposition, a supposition that we will prove to be false for one feature.

Thirdly, the separate class method [48] is evaluated to handle missing data. The separate class method defines a new category to represent the missing data of a feature so that each feature has its own category to represent its misses. In the case of numeric type features, the missing data receive a value that is outside the range of the feature’s values. In this way, the required separation between the missing data and the correct values is created.

Each approach creates a different dataset size with a different number of patient samples and a different number of features per patient. Hence, our comparison selects the best approach in terms of the best training of the ML model. That is the approach that has the best trade-off between the number of samples and the features so that the RF provides the most accurate prediction.

#### Renormalisation

We renormalise the numerical features so that every feature’s different values are separated based on the same scale, which is especially relevant for those techniques such as SVM or KNN that use the notion of distance in a metric space. Hence, all numerical data are rescaled to values in [0,1]. This renormalisation is also applied on the separate classes associated to the missing data, and we assign them the value − 0.5 since there are no negative values in any dataset.

### Machine learning techniques

Three supervised ML classifiers are used: SVM, RF and KNN. We devote the next three sections to briefly presenting the ML techniques.

#### Support vector machine

SVM is a supervised ML technique [51, 52]. In binary classification problems over a dataset of instances of dimension *L* + 1, this technique finds an *L*-dimensional hyperplane that separates the two different classes, maximising the distance of the closest instances in the dataset -called support vectors- to the hyperplane. The distance from the support vectors to the hyperplane is called margin. In other words, SVM finds the hyperplane that maximises the margin of the support vectors. So, as stated above, it requires a definition of distance on the dataset’s features to evaluate the separation between the instances and the hyperplane. The hyperplane is defined by its normal vector, *w*, and the hyperplane equation is *w*^*T*^ ⋅ *x* + *b* = 0 with *w*^*T*^ being the transpose of the normal vector and $\frac {b}{\left \lVert w \right \rVert } $ the offset of the hyperplane from the origin. Equation 2 defines the optimisation problem.

2 $$  \begin{aligned} \min \quad & \left\lVert w \right\rVert \quad \textrm{subject to} \quad y_{i} \cdot (w^{T} \cdot x_{i} + b) \geq 1 \end{aligned}   $$

There are two types of SVM classifiers: linear and nonlinear. In the former, SVM operates on the raw data to find the hyperplane under the supposition that the data are linearly separable, whereas the latter transforms the original instances by adding extra similarity features to try to create a linearly separable dataset under the supposition that the original one was not. The most used similarity function is the Gaussian Radial Basis Function [53]:

3 $$  \phi(x_{i},p) = e^{-\gamma \cdot \left\lVert x_{i} - p \right\rVert^{2}}   $$

where the set of points *p* determines the landscape used to calculate the new features, and *γ* ∈ [0,1] is a regularisation hyperparameter used to control the over- and underfitting of the SVM model.

There are also two types of SVM models depending on whether a few instances of one class are allowed to be located within the margin region or even in the region assigned to the other class. If no instance of one class can be within the margin region or the region assigned to the other class, then a hard margin classification is defined. In any other case, it is a soft margin classification. The soft margin classification allows the misclassification of some instances but provides higher margins in the classification whereas hard margin classification typically provides a clean but narrower margin. In the former case, the SVM has better generalisation capabilities, that is, lower overfitting. SVM implementations provide a hyperparameter to control the softness of the margin, *C*. The higher the *C*, the stricter the classification.

#### Random forest

RF is a supervised ML technique used in both classification and regression [54]. In classification problems, it creates multiple decision trees, each one providing its classification output, and combines the results of all the trees using an aggregation function to provide the classification of the given instance. The potential of this technique is based on the aggregation of weak learners in order to provide high-accuracy predictions. Nevertheless, high accuracy requires the technique to satisfy certain requirements, the first of which is the independence of the individual trees.

In this work, (i) the trees are binary and provide output that can take one of two values, {− 1,1}; (ii) the RF prediction is an aggregation function, i.e. the majority vote, of individual tree predictions; and (iii) independence is achieved by using different subsets of instances to train every individual tree. The sampling of the subsets can be performed using two different schemas: sampling with replacement, called bagging, or without replacement, called pasting. Thus, each individual tree has a larger bias than if it were trained using the complete training set, but the aggregation of trees provides a lower bias-aggregated classification.

The form of a single classification tree is determined by the order in which the features are used to create that tree; that is, in the same set of instances, a different order in the selection of the features used to create the tree generates different trees. One of the most used algorithms to train decision trees is the classification and regression tree (CART). CART splits the training subset into two subsets using a single feature and a threshold for such feature, searching for the tuple feature/threshold that provides the purest subsets. Equation 4 presents the fitness metric used by CART to measure the purity of a node’s classification where *m* is the total number of instances being classified in the node, *m*_left_ and *m*_right_ are the numbers of instances in the left and right splits, respectively, and *G* is the metric that measures the impurity of the splits. The lower the value of *J*, the purer the classification.

4 $$  J = \frac{m_{\text{left}}}{m} \cdot G_{\text{left}} + \frac{m_{\text{right}}}{m} \cdot G_{\text{right}}   $$

Two impurity metrics are commonly used [55]: the Gini impurity, Eq. 5, and the entropy-based impurity, equation 6.

5 $$ \begin{array}{@{}rcl@{}} G & =& 1- {\sum}_{c=1}^{2} {p_{c}^{2}}  \end{array} $$

6 $$ \begin{array}{@{}rcl@{}} E & =& - {\sum}_{c=1}^{2} p_{c} \log(p_{c})  \end{array} $$

where *p*_*c*_ is the ratio of instances of class *c* in the set of instances in the node. Each node only has instances of two classes: bacteraemia or no bacteraemia. For that reason, the sum upper limit is 2.

Finally, the decision tree can be regularised with the following hyperparameter [56]: the maximum depth of the trees, the minimum number of samples in a node to be split, the minimum number of samples of a leaf node, the maximum number of leaf nodes and the maximum number of features to be tested in order to split a node.

#### K-Nearest neighbours

We use the supervised flavour of this simple nonparametric ML technique to classify the binary-class instances [57]. Given a new feature vector, **f**_*l*_, it assigns its class, *y*_*l*_, by finding the *k* of nearest instances in the dataset feature space and combining their classifications (i.e. averaging or voting). So, like SVM, this technique requires the definition of distance in Eq. 1. However, this technique does not need a training phase, and it achieves a very high capacity: the larger the training set, the higher the capacity.

The selection of the value for *k* should follow these rules: (i) the value should be a prime number to avoid ties; (ii) it should be less than the total number of reference instances in an instance class; and (iii) its value should be large enough to avoid false classification caused by outliers. The actual value of *k* is found using a grid search on a range of reasonable values. The technique returns the majority of the *k* nearest neighbours that share the same class. The fine-tuning of this hyperparameter requires it to be searched for using a heuristic.

### Validation

In our experiments, the 10-fold cross-validation approach is followed so that the dataset is divided into ten subsets and each subset is used as a validation set whereas the remaining nine subsets are used for training a model. This procedure is repeated for every subset, so ten models are obtained. The performance of the ML technique is measured as the average performance of the ten models obtained with different training sets and validated on different sets.

## Data analysis

The analysis was performed in Python 3.7 using sklearn 0.23 for model inference and ELI5 0.10.1 for the permutation importance method.

### Data bias

First, we study any bias in the distribution of values in the datasets. As stated at the beginning of this section, datasets contain a balanced percentage of values in the predicted variable: bacteraemia (51.3%) and no bacteraemia (48.7%); the latter includes both actual negative bacteraemias and contaminated cultures.

**Fig. 1:**
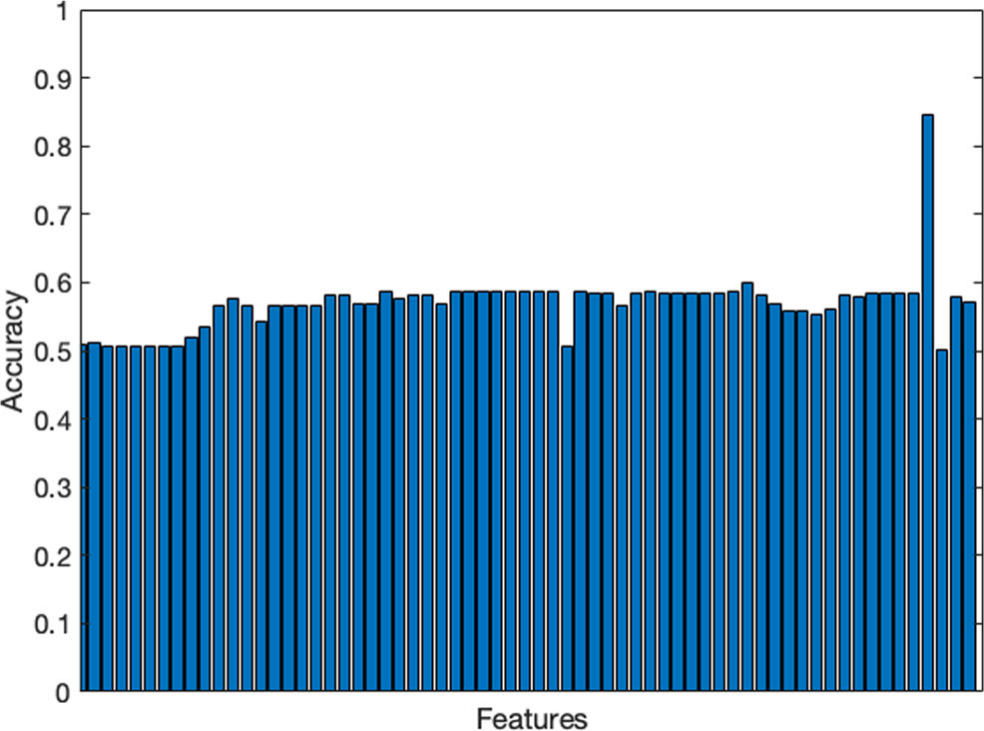
Accuracy of the individual features when only two classes (missing and non-missing) are used to predict bacteraemia

Similarly, we check whether missing data in $\mathbb {F}$ are correlated with the predicted variable. That is, if the MCAR assumption holds for the data. Figure 1 presents the classification accuracy for all the features, one feature at a time, in the dataset.

**Fig. 2:**
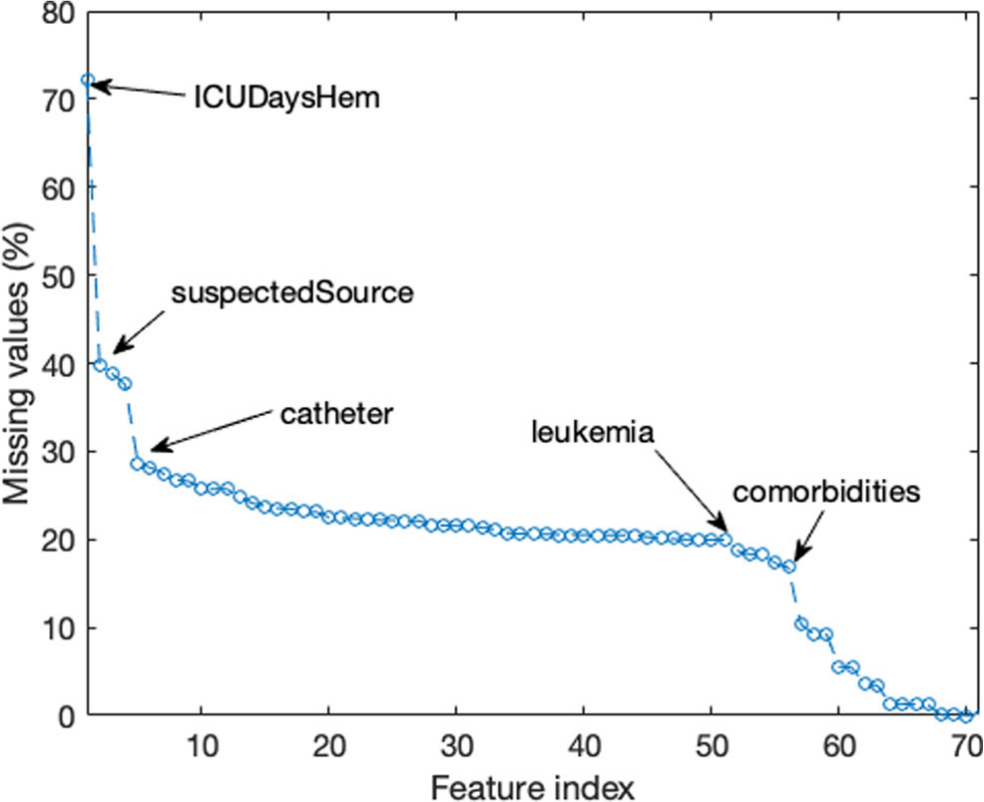
Percentage of missing values for all the features in $\mathbb {F}$. The features are sorted on x-axis as in Table 5. The annotations in the graph mark the inflection points, and they facilitate cross-searching in Table 5

The missing class of the suspected source (the peak in the histogram with an accuracy of 82.6%) is a good predictor of no bacteraemia. In contrast, the remaining features have a slight bias in the prediction. The ratio of missing data for this feature is around 40%, as Fig. 2 illustrates. The feature’s importance, with such a high ratio of missing data, is suspicious and indicates a correlation between the missing-data class and the variable predicted. Hence, 72.4% of the instances with a suspected source, either ‘unknown’ or any organ in the body, are bacteraemia. On the contrary, only 7.2% of the missing suspected sources are bacteraemia.

These figures state a missing at random (MAR) [47] behaviour for this feature. During database generation, the physician, who is typically good at predicting the focus of infection but not so good at predicting which of them are accompanied by bacteraemia, only includes the suspected source in the database once the bacteraemia has been detected. In other words, the physician decides that writing down the source of infection is of no interest for non-bacteraemia cases. This feature is removed from both datasets.

### Missing data

This section presents the number and distribution of missing data per feature. Figure 2 illustrates the percentage of missing values for the features in $\mathbb {F}$. The percentage is above 70% for the worst feature (number of days in ICU previous to culture) and between 40 and 37% for the following three features: the suspected origin of the bacteraemia previous to culture, the results of PCR testing and the source of bacteraemia in the last hospital department. Following them, there are 50 features with missing-data percentages from 30 to 20%.

We evaluate three different approaches to handle the high number of missing data [49]. The complete case data approach removes the instances with missing data to obtain a new dataset without misses. If we apply this approach on our original dataset, then the new dataset only contains 476 complete instances out of 4357. Hence, this approach is inappropriate due to the large volume of data lost. Nevertheless, we evaluated its achievements to classify the bacteraemias accurately.

**Fig. 3:**
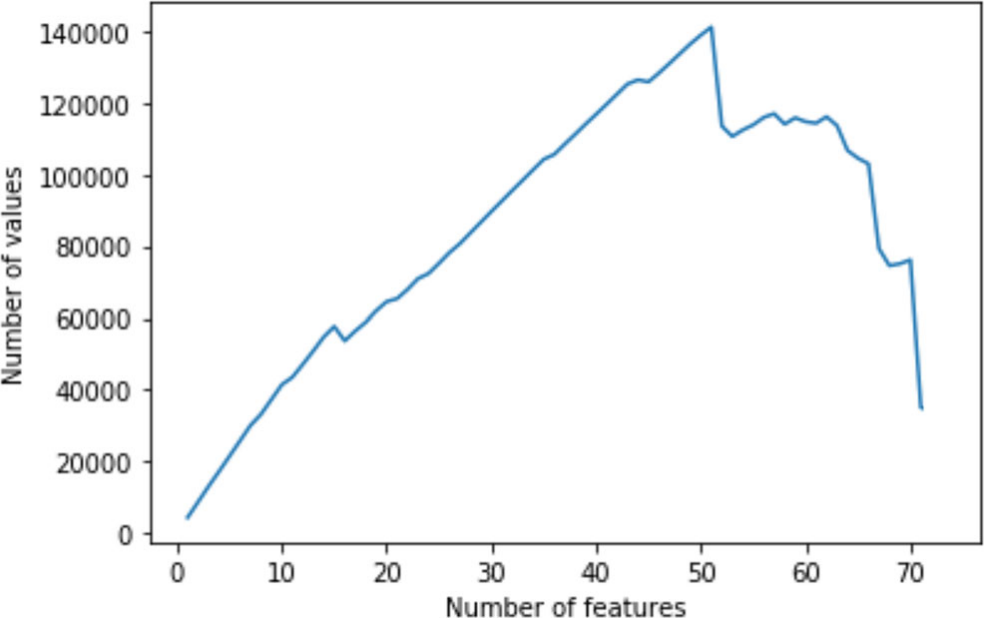
Number of features versus number of non-missing values in dataset

The second approach removes the features with a higher number of missing data. Figure 3 illustrates the evolution of the total volume of data in all complete instances versus the number of complete instances. In our case, the optimal number is 51 features with 2760 instances, totalling 140,760 non-missing values in the dataset. As in the previous approach, we think this is also inappropriate because (i) it removes critical features from datasets such as, for example, the suspected medical source of the patient’s infection, and (ii) it removes 33.8% of the features and 44.6% of the number of instances. Nevertheless, we also evaluated its achievements to classify the bacteraemias accurately.

Thirdly, the separate class method [48] was evaluated to handle missing data. The separate class method defines a new category to represent the missing data of a feature so that each feature has its own category to represent its misses. In the case of numeric type features, the missing data receive a value that is outside the range of the feature’s values. In this way, the required separation between the missing data and the correct values is created.

The performance of the three missing-data methods was compared using RF as the testbench. In these comparisons, the renormalised separate class method obtains the best performance, and for that reason, it is the method of choice in this work.

### Prediction results

The three ML techniques have been evaluated using the same procedure: (i) the dataset is split into 80/20 training/testing sets, (ii) grid-search 10-fold cross-validation is run on training data for the ML techniques to find their best hyperparameters, and (iii) the best hyperparameters are applied on the testing split of the dataset.

#### SVM

The hyperparameters of the SVM model are swept in the ranges *C* = {0.1,0.2,…,1,2,…,10,20,…,100} and $\gamma = \left \{ \frac {1}{L^{\prime }}, \frac {1}{ L^{\prime } \cdot \sigma }, 0.1, 0.2, \ldots ,1 \right \}$ with *σ* being the data variance, by using the Gaussian Radial Basis Function.

The hyperparameters for the best pre_culture SVM model are $\gamma = \frac {1}{L^{\prime }}$ and *C* = 9, which implies that the instances are separable. Table 1 summarises key metrics to evaluate the predictive capacity of the model: accuracy, sensitivity, specificity, positive predictive value (PPV) and negative predictive value (NPV). The average accuracies of the best pre-culture SVM model are 76.9 ± 1.7% in the training phase and 75.9% in the testing phase. Accuracy in the testing phase is only 1.0% lower, proving the good generalisation capabilities of the model. This model has a sensitivity of 80.7% with a specificity of 71.4%, PPV of 72.8% and NPV of 79.6%.

**Table 1:** Accuracy, specificity, sensitivity, positive predictive value (PPV), negative predictive value (NPV) and area under the curve (AUC) of the models

ML	Model	Accuracy (%)		Sensitivity	Specificity	PPV	NPV	AUC
		Training	Testing	(%)	(%)	(%)	(%)	
SVM	pre_culture	76.9 ± 1.7	75.9	80.7	71.4	72.8	79.6	0.85
	mid_culture	83.0 ± 1.4	80.5	81.3	79.7	80.5	80.5	0.88
RF	pre_culture	79.5 ± 1.4	78.2	86.1	70.7	73.6	84.3	0.86
	mid_culture	85.6 ± 1.4	85.9	87.4	84.4	85.2	86.6	0.93
KNN	pre_culture	72.8 ± 2.3	76.5	89.6	65.2	69.0	87.9	0.85
	mid_culture	78.0 ± 2.7	78.4	87.4	69.9	73.6	85.2	0.88

For the sake of saving space, the standard deviation is presented in compact notation

The features’ importance has been evaluated using importance sampling, and the left two columns in Table 2 present the top 10 most important features of this SVM model. Among them, the top 3 to predict bacteraemia are a chronic respiratory disease, the number of days in ICU before blood extraction and the presence of catheters.

**Table 2:**
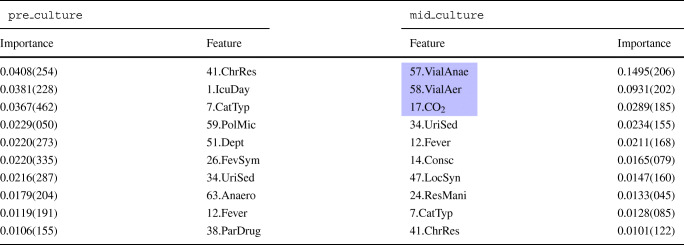
Feature importance for SVM

The left-hand side of the table ranks the top 10 features for the pre_culture model, whereas the right-hand side ranks the top 10 features for the mid_culture model. In blue, the new features included in the mid-culture model. For the sake of saving space, the standard deviation is presented in compact notation, that is, 0.4514(540) ≡ 0.4514 ± 0.0540. The number close to the feature name refers to the Id. in Table 5 that describes the feature

The mid_culture SVM model was designed using the same procedure. In this case, the hyperparameters of the best model are $\gamma = \frac {1}{L^{\prime }}$ and *C* = 8, which implies that the instances are slightly more separable than in the pre-culture dataset. The average accuracy of the training phase is 83.0 ± 1.4% and the testing phase achieves an overall accuracy of 80.5%, sensitivity of 81.3%, specificity of 79.7%, PPV of 80.5% and NPV of 80.5%. The usage of intermediate results of the blood culture increases all the metrics from 5 to 8%. Table 2 illustrates the most relevant features to predict bacteraemia using the importance sampling method. According to this table, three out of the four new features rank in the top 5 most relevant features: growth in anaerobic and aerobic vials, and the number of days until CO_2_ detection.

**Fig. 4:**
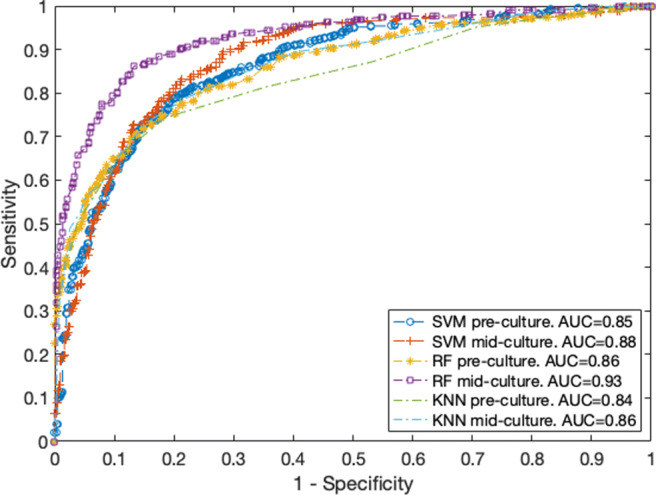
ROC for the best SVM, RF and KNN for models

Figure 4 presents the ROC of the three ML techniques evaluated for the two datasets. The mid_culture SVM ROC has an area under the curve (AUC) of 0.88, performing better than the pre-culture SVM model, which has an AUC of 0.85.

#### RF

We have not constrained either the maximum depth, the minimum number of samples in a node or any other of the hyperparameters stated in “Random forest”, and we use the Gini impurity metric. The only hyperparameter of the model evaluated in the grid-search exploration is the number of trees, which is found in {1,2,…,90}.

The best pre_culture RF model averages an accuracy of 79.5 ± 1.4% in the grid-search 10-fold cross-validation with 86 trees, and an accuracy of 78.2% during the testing phase. As for SVM models, the variation in accuracy refutes the overfitting of the model. Table 1 summarises the key metrics that clinical practitioners use to evaluate the models’ predictive capacity. The features’ importance has been evaluated using the permutation importance algorithm, and Table 3 presents the most critical features of the model.

**Table 3:**
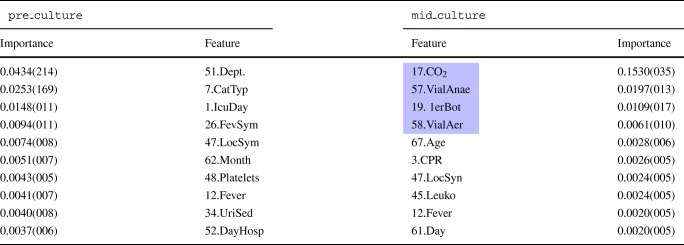
Feature importance for RF

The left-hand side of the table ranks the top 10 features for the pre_culture model whereas the right-hand side ranks the top 10 features for the mid_culture model. In blue, the new features included in the mid-culture model. The number close to the feature name refers to the Id. in Table 5 that describes the feature

The mid_culture RF model uses 68 trees and obtains an average accuracy of 85.6 ± 1.4% in the training phase and reduces the size of the RF model by 34.9%. This model performs better than the pre-culture one, improving all the predictive metrics: it increases accuracy 6.1% in the training phase -a value similar to that observed in SVM models- and 7.7% in the testing phase -an improvement higher than that observed in the SVM models-, sensitivity by 1.3%, specificity by 13.7%, PPV by 12.6% and NPV by 2.3%.

Table 3 illustrates the most critical features to predict bacteraemia for this model. As for the SVM models, the new features are ranked among the top ones. Hence, the top-ranked feature is the number of days at CO_2_ detection followed by the positive in anaerobic vials, the first blood culture vial with growth and the positive in aerobic vials. Regarding the distribution of values in the rankings, the two RF rankings are more unbalanced than the SVM ones, with an outstanding feature in both cases, which doubles the importance of the second feature in the pre-culture model and which is 8 × for the mid-culture model.

#### KNN

The only hyperparameter for this classifier is *k* which, in this study, is found in {1,2,…,20}.

The best pre_culture KNN model uses *k* = 15 neighbours, and the best mid_culture model uses *k* = 9. Table 1 summarises the key metrics to evaluate the predictive capacity of the KNN models. The best pre-culture KNN model averages an accuracy of 76.5% during the testing phase. As in previous models, the inclusion of mid-culture features improves the KNN model’s performance, although less significantly -only a 1.9% increment in testing accuracy- and it even has a slight decrease of 2.2% in sensitivity and of 2.7% in NPV. Moreover, similar to RF models, the inclusion of new features reduces the size of the model, in this case the number of relevant neighbours.

Table 4 presents the top 10 most important features in the KNN model according to importance sampling criteria.

**Table 4:**
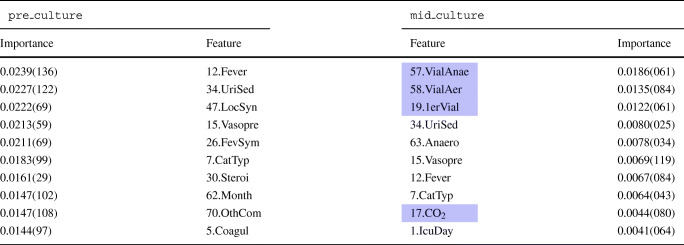
Feature importance for KNN

The left-hand side of the table ranks the top 10 features for the pre_culture model whereas the right-hand side ranks the top 10 features for the mid_culture model. In blue, the new features included in the mid-culture model

Finally, Fig. 4 graphs the ROC of the two KNN models with AUCs of 0.85 and 0.88. Hence, this technique has a predictive power lower than the previous ones.

## Discussion of the results

### Data interpretation

Typically, medical records contain missing data that can bias the conclusions of the ML techniques. The separate class method provides a mechanism to handle the missing data, preserving the number of patients in the study and providing good metrics in the classifiers. We did not evaluate imputation methods based on ML algorithms, such as KNN, to predict the missing values in the training data because they can infer relationships among the features that could distort the data structure [58] or such as the more efficient imputation method missForest [59] because this iterative imputation method must be run with every single new patient, which would increase the computational cost of every new prediction when the system is in production.

The importance rankings of the three ML techniques provide a significant ratio of common top features for both datasets. Hence, for the pre-culture models, the number of days in ICU before blood culture extraction, the presence of catheters, fever and the presence of symptoms related to the source of fever and the presence of urine sediments are critical features of major importance. The month of the blood culture appears for the pre-culture KNN and RF models. Hence, both techniques detect seasonality in the bacteraemia, although it has a low importance in both techniques.

Regarding the models for the mid_culture dataset, the new features in this dataset are the most important for an accurate prediction of the bacteraemia, displacing the top features of the pre_culture model. Indeed, their importance in the model exceeds the importance of all the features in the pre_culture model. In particular, the mid_culture RF model ranks the four new features among the top of the ranking, whereas the other two techniques only include three out of the four new features.

This consistency highlights that prediction capability is a characteristic intrinsically related to the data already available in most of the hospital health records.

The feature importance for the pre_culture SVM and KNN models is balanced. The top 3 feature importances are within a range of 10.0% of the most important one, and then the importance is reduced softly for the remaining seven features. The high number of features taken into account for the models to generate a prediction justifies physicians’ difficulty in generating accurate predictions: they cannot handle such a large number of variables. In particular, the two KNN rankings are the most balanced of the three ML techniques. The first five features in the pre-culture model and the first three features in the mid-culture model have very similar values, although the dispersion of accuracy in the training stage doubles the dispersion values of the other ML techniques, which justifies why the KNN technique produces less predictable accuracy for the model.

On the other hand, the feature importance of the pre_culture RF model is less balanced, with a critical feature then two less relevant features, and the remainder are mostly irrelevant. This behaviour is exacerbated in the mid-culture model in which new features dominate the classification. For this reason, in the presence of these features, the physician could make a prediction based on a lower number of features. Nevertheless, the features, as stated above, coincide in almost half of the cases.

The test accuracy of the ML techniques on the pre_culture dataset ranges between 75.9% for SVM and 78.2% for RF. These values are increased by around 9.8% when using the new features in the mid-culture dataset, with mid-culture RF model obtaining an accuracy of 85.9% . Hence, the accuracy of ML techniques is 8 × human accuracy (from 3.6 to 10% according to [22]).

Regarding the key metrics to evaluate the predictive capacity of the model, their values range from 80.7 to 89.6% for sensitivity, 65.2 to 84.4% in specificity, 69.0 to 85.2% for PPV and 79.6 to 86.6% for NPV, with the mid-culture RF model outperforming the other models and achieving an average accuracy of 85.9 ± 1.4%, sensitivity 87.4%, specificity 84.4%, PPV 85.2%, NPV 86.6% and an outstanding AUC of 0.93 with improvements of 6.7% with regard to the accuracy of the second best technique, SVM, 6.1% in sensitivity, 4.7% in specificity, 4.7% in PPV and 6.1% in NPV.

AUC is above 0.85 for all models, and the presence of the new features increases the AUC from 3.5 to 8.1% with respect to the pre_culture AUCs. A predictive model in the medical practice must have an AUC greater than 0.7, and a good predictive model has AUC≥ 0.8. The previous results in the literature using classical modelling techniques in specific types of bacteraemia are as follows: pneumonia [32] with AUC 0.79, skin-related [33] with AUC 0.71 or any type [34] with AUC 0.77. Therefore, the ML values of AUC, sensitivity, specificity, predictive positive and negative values exceed the results described in the literature.

Previous results indicate that bacteraemia prediction can be achieved using already available hospital records with better figures of merit than the physicians’ predictions. These predictions can help physicians make an appropriate diagnosis and prevent complications, where, in this context, ‘appropriate’ means both in time, i.e. as soon as possible, and in type, with the more specific and personalised antibiotics and treatment for each patient.

### Interplay between COVID-19 and bacteraemia

Nowadays, we are experiencing the COVID-19 pandemic, so it is necessary to refer to the possible association between COVID-19 and bacteraemia and the utility of ML techniques in this kind of patient. In this context, bacteraemia is rare for COVID-19 patients, which supports the judicious use of blood cultures in the absence of compelling evidence for bacterial co-infection [60]. In some reports, bacteraemia with *S. aureus* is associated with high mortality rates in patients hospitalised with COVID-19. *S. aureus* infections are a known complication of other viral pandemics, such as the Spanish flu in 1918–1919 and the H1N1 influenza pandemic in 2009–2010, suggesting that the interaction of *S. aureus* with SARS CoV-2 is similar to that in influenza [61]. The proposed mechanisms of viral-induced bacterial co-infections include the viral modification of airway structures, as well as the initiation of immune-suppressive responses [62]. A similar mechanism has been described in another report of oral infections where the authors suggest that poor oral hygiene and periodontal disease could produce the aggravation of COVID-19 [63].

Secondary bacteraemia has been developed in 37% (27/73) of patients with acute respiratory distress syndrome [64]. However, it has not been defined whether bacteraemias were secondary to pneumonia or typical hospital-acquired infection.

In this sense, ML techniques could help physicians predict bacteraemia as a secondary infection in COVID-19 patients, mostly in critical COVID-19 patients, who suffer these secondary infections more frequently [65].

## Conclusions and recommendations in the framework of 3P medicine

### Conclusions

The three ML supervised classifiers create accurate predictive models of the blood culture outcome using hospital electronic health records, i.e. data previous to blood extraction and data measured in the first hours/days of the blood culture. The concordance in the results of the three classifiers increases the power of the conclusions and confirms the viability of ML techniques as a key technology for applying the PPPM/3PM principles to improving patients’ survival rates significantly and providing more cost-effective management of the disease.

### Expert recommendations

Bacteraemia is an entity with high morbidity and mortality. Its early diagnosis and an appropriate early antibiotic treatment are critical. For these reasons, in this kind of pathology, it is essential to combine predictive techniques and personalised treatments in which ML techniques can help physicians diagnose, reduce time to treatment and manage bacteraemia. ML techniques could help determine preventive actions to avoid this entity, and secondly, to optimise the cost of the disease. If physicians could predict bacteraemia, then they could avoid the intervention to obtain blood samples, the use of four to six bottles for blood culture per patient, the time lapse devoted to the culture and the procedures to identify possible contaminant microorganisms with their associated cost in time and money.

Regarding the selection of antibiotic treatment and its duration, both could change depending on whether the patient is suffering from bacteraemia or not. Usually, diseases associated with bacteraemia need a longer duration of antibiotic treatments. This duration could be optimised if physicians could predict whether a patient has or does not have bacteraemia. If we could shorten the duration of antibiotic treatment, we would spend less money on each patient and avoid secondary effects associated with longer antibiotic treatment, such as antibiotic resistance [66].

Therefore, continuous data extraction from electronic medical records could help physicians identify bacteraemia and the progression to a severe disease earlier and provide timely interventions, such as appropriate antibiotic treatment, to reduce mortality and morbidity [67, 68].

The adoption of ML technologies in the framework of 3P medicine depends entirely on the accuracy of their models, which is related to the availability of datasets with low missing value rates and no bias in the missing values because of the physician’s a priori interpretation of the data. Patient databases play a central role in 3P medicine [1], and it is critical to ensure their completeness and avoid depending on the physician’s discretion at the time of completing the database records. This requirement should be included in database design specifications and the design of database user interfaces.

The application of ML techniques also depends on the availability of structured datasets. Most hospital records store health information according to the European Commission’s Recommendation on Electronic Health Records [69], but data would have to be stored in a format suitable for the automatic manipulation of the features, avoiding as much as possible those features expressed in natural language that hinder the extraction of structured information.

Predictive models play a key role in bolstering decision systems, and ML techniques have outstanding potential to create models with an excellent level of accuracy [70]. They have been used to identify useful correlations between biometric, genetic and environmental data with the potential risks and benefits of certain therapeutic choices [71]. They also have great potential to exceed the performance of physicians’ heuristics, reducing lags in diagnosis and treatment costs when their application is extended from the genomic and biometric data to the clinical and demographic data in the patient’s records.

Our future work will focus on studying non-structured features (medical texts described in natural language), also included in the database, that could improve the model’s accuracy. Additionally, we will validate these findings using independently collected databases and, subsequently, under regulatory approval, we will develop an app for mobile devices that enables the translation of these results to the hospital practice by providing a prediction to the physician at the bedside based on the latest available patient records.

These ideas are directed to improve predictive and personalised treatment in a disease as bacteraemia that currently continues producing a high level of mortality.

## Data Availability

The code used in this study is available from the corresponding author on reasonable request.

## Appendix A: Features in the study

Table 5 presents the description of the features used in this work.

**Table 5:** Features in the study sorted according to the number of missing values

#	Description
1	Days in Intensive Care Unit before blood culture extraction
2	Suspected source of bacteraemia previous to blood culture
3	C-reactive protein level
4	Days after last catheter was placed
5	Altered coagulation values
6	Heart rate
7	Catheter type
8	Urea (mg dl^− 1^)
9	Diastolic blood pressure
10	Systolic blood pressure
11	Hypotension
12	Fever. Armpit temperature> 38 ^∘^C at the time of blood extraction
13	Armpit temperature at blood extraction in Emergency Room
14	Consciousness level at the moment of bacteraemia
15	Use of vasopressor agents at the time of bacteraemia
16	Cardiorespiratory resucitation at the moment of bacteraemia
17	Days to CO_2_ detection
18	Days with fever before blood culture is obtained
19	First blood culture vial with growth
20	Genitourinary manipulations
21	Vascular manipulations
22	Thrombocytopenia
23	Leukocytosis
24	Respiratory manipulations
25	Digestive manipulations
26	Symptoms related to the source of fever
27	Glycemia
28	Neutropenia
29	Previous surgery
30	Steroids
31	Immunosuppressants
32	Drug addiction
33	Urine sediment
34	Blood creatinine (mg dl^− 1^)
35	Comorbidites by Weinstein classification
36	Alcoholism
37	Renal insufficiency
38	Intravenous drug addiction
39	Cardiopathy
40	Diabetes
41	Chronic respiratory disease
42	Hepatopathy
43	Active neoplasia
44	Hospitalization longer than 48h in last 12 months
45	Leukocytes (*μ**l*^− 1^)
46	Polymorphonuclear leukocytes (%)
47	Syndromes related to the source of fever
48	Platelets (*μ**l*^− 1^)
49	Hospitalization in the last 30 days
50	Hemoglobin (gdl^− 1^)
51	Specialty where bacteraemia is suspected
52	Days in Hospital before blood extraction
53	Antibiotics
54	Systematic urine analysis
55	Comorbidities
56	Number of blood culture vials obtained
57	Growth at least in anaerobic environments
58	Growth at least in aerobic environments
59	Polymicrobial bacteraemia microorganisms
60	Growth medium of true bacteraemias
61	Day of blood extraction
62	Month of blood extraction
63	Anaerobic bacteraemias versus other bacteraemias
64	Fungal bacteraemias versus other bacteraemias
65	Anaerobic microorganisms
66	Polymicrobial origin bacteraemia
67	Age
68	Gender
69	Final classification of blood culture

## Data availability

The dataset is available from the corresponding author on reasonable request.
